# Sex-related differences in periprosthetic joint infection research

**DOI:** 10.5194/jbji-9-137-2024

**Published:** 2024-04-30

**Authors:** Domenico De Mauro, Cesare Meschini, Giovanni Balato, Tiziana Ascione, Enrico Festa, Davide Bizzoca, Biagio Moretti, Giulio Maccauro, Raffaele Vitiello

**Affiliations:** 1 Department of Orthopedics and Rheumatological Sciences, Fondazione Policlinico Universitario A. Gemelli IRCCS, Rome, Italy; 2 Department of Orthopedics and Geriatric Sciences, Catholic University of the Sacred Heart, Rome, Italy; 3 Orthopedics and Traumatology Unit, Department of Public Health, Orthopedic Unit, “Federico II” University, Naples, Italy; 4 Service of Infectious Diseases, AORN Antonio Cardarelli Hospital, Naples, Italy; 5 Orthopedics and Traumatology Unit, UOSD Vertebral Surgery, AOU Consorziale “Policlinico”, Bari, Italy

## Abstract

**Introduction**: Periprosthetic joint infections (PJIs) have emerged as a focal point in the realm of orthopedics, garnering widespread attention owing to the escalating incidence rates and the profound impact they impose on patients undergoing total joint arthroplasties (TJAs). Year after year, there has been a growing trend in the analysis of multiple risk factors, complication rates, and surgical treatments in the field. This study aims to illuminate the status of the sex-related differences in periprosthetic joint infections and advance research in this field. **Methods**: A systematic review was carried out following the Preferred Reporting Items for Systematic Review and Meta-Analyses (PRISMA) guidelines. The final reference list comprised longitudinal studies (both retrospective and prospective) and randomized controlled trials. A sex-based analysis was conducted to assess differences between males and females. **Results**: A total of 312 studies were initially identified through online database searches and reference investigations. Nine studies were subsequently included in the review. Eight out of nine studies examined the risk of developing PJI after total joint replacement. Notably, only half of these studies demonstrated a statistically significant value, with a p value <0.05 , indicating a higher risk of infectious complications in males compared to females. **Conclusion**: According to the current literature, there appears to be a propensity for males to develop periprosthetic joint infection after total joint arthroplasty at a higher rate than the female population. Enhancing sex-related analysis in this field is imperative for gathering more robust evidence and insights.

## 1 Introduction

Periprosthetic joint infections (PJIs) have emerged as a focal point in the realm of orthopedics, garnering widespread attention owing to the escalating incidence rates and the profound impact they impose on patients undergoing total joint arthroplasties (TJAs) (Balato et al., 2019b). As the joint replacement procedures proliferate globally, the prevalence of PJIs rise in tandem, presenting a parallel challenge (Rovere et al., 2021; Dudareva et al., 2021). This surge in PJI cases exerts a substantial strain on healthcare systems worldwide, resulting in elevated economic and social burdens (Premkumar et al., 2021). Patients afflicted by PJIs endure a tangible decline in joint function and overall quality of life (Nabet et al., 2022). The ramifications extend beyond physical discomfort, impacting daily activities and mobility. The psychological toll, marked by persistent pain and treatment uncertainties, adds to the burden. As joint function worsens, so does the ability to enjoy once-favored activities, contributing to an evident deterioration in overall quality of life (Jenny et al., 2013).

The spotlight on periprosthetic joint infections has significantly intensified in recent years, fueled by a surge in research papers and studies over the last decade. According to the current literature, hip and knee PJIs represent the most prevalent occurrences in the daily practice of orthopedic surgeons, each exhibiting a similar incidence rate of approximately 2 % as reported by the US national register (Tande and Patel, 2014). Subsequently, shoulder PJIs follow, with an incidence rate of 1.1 %. Distinct considerations arise for elbow PJIs, where the incidence rate is notably higher, at 3.3 %. This elevated rate is likely attributed to the substantial number of arthroplasties performed in patients with rheumatoid arthritis, coupled with the challenges posed by the relatively poor soft-tissue coverage around the implant in this anatomical region (Tande and Patel, 2014).

This underscores the prominent role of PJIs in arthroplasty revisions, constituting approximately 15 % of hip revisions and exceeding a quarter, specifically 25.5 %, of knee revisions (Inabathula et al., 2018).

Year after year, there has been a growing trend in the analysis of multiple risk factors, complication rates and surgical treatments in the field (El Ezzo et al., 2020; Bouji et al., 2022).

However, it is noteworthy that gender considerations have frequently been confined solely to demographic data, lacking more in-depth analysis in many instances. This tendency to relegate gender to a mere demographic aspect underscores the need for a more nuanced exploration of its role in the various aspects of risk factors, complications and treatment outcomes (Basilico et al., 2020; McCulloch et al., 2022).

Despite this body of work, a notable gap persists in the analysis of PJI's impact on male and female patients, potentially giving rise to a sex-related disparity in PJI research.

Understanding how PJI affects male and female patients differently is crucial for tailoring interventions and optimizing outcomes (Gooding et al., 2011), bridging this gap in research would not only contribute to a deeper understanding of PJI but would also pave the way for gender-sensitive strategies in prevention, diagnosis and treatment.

This study aims to illuminate the current status of the gender gap in periprosthetic joint infections and to advance research in this field. This will be achieved through a comprehensive narrative review of the existing literature. By synthesizing and analyzing the available works, the study aims to provide an up-to-date understanding of how sex-related differences impact the incidence, presentation and outcomes of PJI.

## 2 Materials and methods

### 2.1 Study design and eligibility criteria

A systematic review was carried out following the Preferred Reporting Items for Systematic Review and Meta-Analyses (PRISMA) guidelines (Page et al., 2021) up to January 2024. Research of papers investigating sex-related differences in periprosthetic joint infections was performed in different online databases: MEDLINE, Scopus and Web of Science. The keywords were combined as follows: (((prosthetic) AND (joint)) AND (infection)) AND ((gender) OR (male) OR (female)). All English-language articles, regardless of publication date, were considered for inclusion in this study. Additionally, the reference lists of selected articles were reviewed to identify any relevant studies missed in the initial database search. The final reference list comprised longitudinal studies, both retrospective and prospective. Exclusion criteria were applied to maintain focus, excluding case reports, expert opinions, prior systematic reviews, letters to the editor and studies not directly related to the review topic.

### 2.2 Study assessment and data extraction

Initially, two independent reviewers (DDM and CM) conducted a screening of titles and abstracts of the studies. Full texts were obtained for abstracts that met inclusion criteria or had any uncertainty. Subsequently, two additional independent reviewers (RV and GB) assessed each study against inclusion criteria, and any discrepancies were resolved through evaluation by the senior author (GM). Data extraction involved recording the participant demographics, risk factors and male–female ratios from each study. The methodological quality of the studies considered for this literature review was evaluated using the Methodological Index for Non-Randomized Studies (MINORS) score. For non-comparative and comparative studies, the MINORS score yields maxima of 16 and 24, respectively (Slim et al., 2003). Two authors (EF and CM) independently determined the MINORS score; the final score was obtained through consensus.

### 2.3 Statistical analysis

The data about periprosthetic joint infections, male–female ratios and the associated risk factors underwent thorough review and collection. Statistical significance was set at a p value of <0.05 . The SPSS software program (SPSS, Inc., Chicago, IL, USA) was employed for tabulating the acquired data. Categorical variables are reported as frequencies and percentages, while continuous variables are presented as means and standard deviations. Precision in reporting was maintained at one decimal digit, with rounding-up applied as needed.

## 3 Results

**Figure 1:**
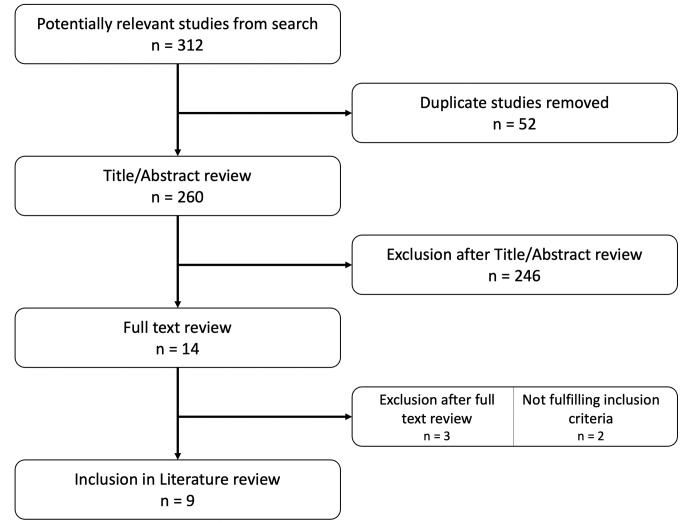
Flowchart according to PRISMA guidelines (Page et al., 2021).

A flow diagram illustrating the search strategy is presented in Fig. 1. A total of 312 studies were initially identified through online database searches and reference investigations. Following the removal of duplicates and the initial screening based on titles and abstracts, the search narrowed down to a final list of 14 papers for in-depth analysis at the full-text level. Nine studies (Browning et al., 2022; Lenguerrand et al., 2019; Tsaras et al., 2012; Walocha et al., 2023; Massin et al., 2016; Tayton et al., 2016; Keemu et al., 2023; Castano-Betancourt et al., 2018; Wimmer et al., 2016) were subsequently included in the review.

The selected studies originated from diverse geographic locations, with contributions from Europe (Lenguerrand et al., 2019; Massin et al., 2016; Keemu et al., 2023; Wimmer et al., 2016), the US (Tsaras et al., 2012; Walocha et al., 2023) and Oceania (Browning et al., 2022; Tayton et al., 2016), with only one other country represented (Brazil) (Castano-Betancourt et al., 2018). The study encompassed a total of 748 569 patients, with data derived from various sources, including national arthroplasty registers. Within this cohort, a total of 5576 cases of periprosthetic joint infections were recorded. In terms of patients' sex distribution, the male population in the PJI group constituted 51.3 %, while the female population accounted for 49.7 %. A summary of these data is presented in Table S1.

In eight out of nine studies (Lenguerrand et al., 2019; Tsaras et al., 2012; Walocha et al., 2023; Massin et al., 2016; Tayton et al., 2016; Keemu et al., 2023; Castano-Betancourt et al., 2018; Wimmer et al., 2016) that examined the risk of developing periprosthetic joint infection after total joint replacement, primarily total knee arthroplasty (TKA), a sex-based analysis was conducted to assess differences between males and females. The studies evaluated the odds ratio (OR) and risk ratio (RR) with a 95 % confidence interval. Notably, only half of these studies demonstrated a statistically significant value, with p value <0.05 (Lenguerrand et al., 2019; Walocha et al., 2023; Tayton et al., 2016; Keemu et al., 2023), indicating a higher risk of infectious complications in males compared to females (single values are shown in Table S2). The analysis spanned various follow-up durations, ranging from 6 months to 5 years, consistently revealing a statistically significant p value in favor of a higher risk of infectious complications in males across these different time points. This highlights a potential sex-related disparity in the susceptibility to PJI after total joint replacement, particularly in the male population. In the study by Browning et al. (2022), the focus was not on analyzing the difference in periprosthetic joint infection risks between men and women. Rather, the study highlighted a statistically significant difference in negative culture infections between males and females. Specifically, the findings indicated a high incidence of negative culture infections in females as compared to males.

## 4 Discussion

Infections emerge as a formidable concern among the complications of prosthetic surgery, exhibiting a variable incidence ranging from 1 % to 2 %. The substantial economic and social impact associated with these infections underscores their significance (DeKeyser et al., 2020). Periprosthetic joint infections have substantial consequences for patients' quality of life, necessitating reinterventions or extended courses of antibiotic treatment (Balato et al., 2019a).

The literature contains numerous studies exploring factors associated with an elevated risk of developing periprosthetic joint infection. However, only a limited number of investigations have specifically explored whether sex may be one such factor.

Sex- and gender-related factors significantly impact health status across the lifespan, influencing disease pathogenesis, responses to pharmacologic and surgical interventions as well as clinical outcomes (Solarino et al., 2022).

Sex pertains to the biological distinctions between males and females, encompassing reproductive or sexual anatomy, hormone levels, gene expression and cyclic variations, which manifest in diverse physiological and anatomical characteristics. Gender is a multifaceted and intricate concept involving various non-biological factors such as educational attainment, sociocultural disparities, psychological dimensions, economic standing, medication use, lifestyle choices, co-morbidities and religious beliefs (Bizzoca et al., 2023). The current orthopedic literature has highlighted a paucity of work focusing on gender- and sex-related factors in the study of PJIs.

In eight of the nine studies scrutinized, the male gender exhibited an association with an elevated risk of periprosthetic joint infection. However, this observation may obscure factors intertwined with daily life habits. For instance, Keemu et al. (2023) assessed various factors contributing to the need for revision due to periprosthetic infection in the Finnish community and identified the male sex as a risk factor for revision. However, this association may be subject to bias. As shown by Keemu et al. (2023), in Finland, the male population tends to engage in smoking and consume substantial amounts of alcohol more than women, behaviors that may predispose individuals to the development of PJI and consequently introduce confounding variables into the analysis.

In the New Zealand registry study, Tayton et al. (2016) highlighted the predisposition of male individuals to the development of periprosthetic joint infection. The authors, however, raised inquiries regarding the potential association with genetic factors, suggesting a more probable link to behavioral aspects, including smoking, dietary habits, hygiene practices or a higher likelihood of presenting earlier with symptoms. While sex stands as one of several risk factors linked to the development of periprosthetic joint infection (Mocini et al., 2021), DeKeyser et al. (2020) highlighted the potential influence of healthcare system variables, particularly in an insurance-based system like that in the US. The authors suggested that sex may be a risk factor associated with the development of PJI in patients with Medicaid insurance. Such individuals often face delayed access to care, presenting with a more advanced disease state. Furthermore, surgeries in Medicaid patients may receive lower reimbursement, potentially leading to the use of less expensive implants, expedited surgery or greater autonomy for less experienced staff. An intriguing avenue for further exploration would be to assess whether, in a national healthcare system such as the Italian one, patients who would be analogous to Medicaid patients in the US exhibit a higher risk of PJI compared to the broader population.

Walocha et al. (2023) describe the male sex as a risk factor for shoulder PJI, associating it with distinct male skin anatomy. They note that males typically have a higher concentration of sebaceous glands in the shoulders and chest, potentially leading to an increased risk of surgical site contamination with *C. acnes* compared to women. However, this review does not document whether there is a genetic factor specifically related to the higher risk of PJI in males. The authors hypothesize that lifestyle habits or anatomical aspects could be factors associated with the male gender's increased susceptibility to shoulder PJI.

The identification of the male gender as a risk factor for periprosthetic joint infection has been consistently reported by Ren et al. (2021) and Resende et al. (2021). Both systematic reviews also highlight that the female gender acts as a protective factor against the development of periprosthetic infections. Ren et al. (2021) highlight that the female gender may be a protective factor against PJI after THA after a long follow-up duration ( ≥3 years) and attribute the higher risk of periprosthetic infections in males to a greater incidence of unfavorable behavioral factors, potentially contributing to this elevated risk. On the other hand, Resende et al. (2021) confirm the higher risk of developing PJI in males undergoing TKA, but interestingly they observe that the female gender is a risk factor for the development of PJI in THA.

However, no specific factor has been identified to explain the different sex-associated risks, likely owing to confounding factors such as behavioral considerations. This aspect remains unclear and warrants further investigation by future studies.

The risk of periprosthetic infection extends beyond the primary implant, persisting even after one-stage or two-stage surgical treatments. In a study by Triantafyllopoulos et al. (2017), it was found that the female gender is correlated with a higher risk of treatment failure and infection recurrence in this context. The potential association with this increased risk in females could be linked to differences in fat distribution and hormonal profiles. However, as of yet, no specific sex-associated characteristic has been definitively identified that could explain this observed result. Further research is needed to delve into the underlying factors contributing to the increased risk of treatment failure and infection recurrence in females undergoing such procedures.

Our review encounters several limitations. The variable of sex, although typically included in demographic data, lacks an in-depth assessment of its relationship with periprosthetic joint infections, thus complicating information retrieval. The challenge lies in the difficulty of locating all relevant studies explicitly describing sex as a risk factor, given that it is often not a primary or secondary endpoint, leading to its omission from titles, keywords or abstracts. Additionally, reliance solely on a literature review may not suffice to generate conclusive evidence in this field; studies with higher levels of evidence are deemed necessary for a more comprehensive understanding.

## 5 Conclusion

According to the current literature, there appears to be a propensity for males to develop periprosthetic joint infection after total joint arthroplasty at a higher rate than the female population. It is noteworthy, however, that the existing literature lacks a sufficient number of studies specifically analyzing this aspect. Furthermore, the sex-related differences in this research field have not been deeply explored. Enhancing gender analysis in this field is imperative for gathering more robust evidence and insights regarding the potential sex and gender-based disparities in the development of PJI after TJA.

## Supplement

10.5194/jbji-9-137-2024-supplement The supplement related to this article is available online at: https://doi.org/10.5194/jbji-9-137-2024-supplement.

## Data Availability

The datasets used and/or analysed during the current study are available from the corresponding author upon reasonable request.
