# Supplementary material for: Sex-related differences in periprosthetic joint infection research
Source: J Bone Jt Infect. 2024 Apr 30;9(2):137–42. doi: 10.5194/jbji-9-137-2024 (PMC11184614; doi:10.5194/jbji-9-137-2024)
Supplement: The supplement related to this article is available online at: https://doi.org/10.5194/jbji-9-137-2024-supplement. [file jbji-9-137-supplement.zip › jbji-9-137-2024-supplement-title-page.pdf]

Supplement of J. Bone Joint Infect., 9, 137–142, 2024
https://doi.org/10.5194/jbji-9-137-2024-supplement
© Author(s) 2024. CC BY 4.0 License.

Supplement of

Sex-related differences in periprosthetic joint infection research

Domenico De Mauro et al.

Correspondence to: Raffaele Vitiello (raffaele.vitiello@guest.policlinicogemelli.it)

- jbji-9-137-2024-supplement-title-page.pdf
- Table S1.pdf
- Table S2.pdf

The copyright of individual parts of the supplement might differ from the article licence.
