# Supplementary material for: Sex-related differences in periprosthetic joint infection research
Source: J Bone Jt Infect. 2024 Apr 30;9(2):137–42. doi: 10.5194/jbji-9-137-2024 (PMC11184614; doi:10.5194/jbji-9-137-2024)
Supplement: The supplement related to this article is available online at: https://doi.org/10.5194/jbji-9-137-2024-supplement. [file jbji-9-137-supplement.zip › Table S2.pdf]

**Article**	**Joint**	**PJI patients**	**Male%**	**PJI risk (M vs. F)**	**p**	**Other data (M vs. F)**	**p**
Browning, 2022	Knee, Hip, Shoulder	55	41,8%	-		NC: 32 (58.2 %) vs. **245** (41.2 %)	**0.016***
Lenguerrand, 2019	Knee	3.659	57,3%	RR: **1.48** (1.41–1.54) vs. 0.82 (0.78–0.86)	**<0.0001***	**-**	
Tsaras, 2012	Knee, Hip	70	38,6%	RR: 1.02 (0.66–1.59) vs. 0.74 (0.52–1.08)	>0.05	-	
Walocha, 2022	Shoulder	879	47,7%	OR CI95%: **2.10** (1.81-2.43) male at 2yrs	**<0.0001***	-	
Massin, 2016	Knee	285	47,0%	OR IC95%: 0.7 (0.4-1.2) male	>0.05	-	
Tayton, 2016	Knee	106	63,2%	OR CI95%: **1.85** (1.24-2.74) male at 1yrs	**<0.05***	-	
Keemu, 2023	Knee	484	54,0%	HR CI95%: **0.5** (0.4–0.6) male	**<0.05***	-	
Castano-Betancourt, 2018	Knee, Hip	38	n.a.	HR CI95%: 1.30 (0.65–2.60) female	0.460	-	
Wimmer, 2016	Knee, Hip	n.a.	n.a.	n.a.	0.133	-	

Table S2 Summary of Risk assessment of developing PJI in patients undergoing TJ, based on gender (M vs. F).

n.a. not available; OR odds ratio; RR risk ratio; CI Confidence Interval; NC negative culture infections
