# Supplementary material for: Sex-related differences in periprosthetic joint infection research
Source: J Bone Jt Infect. 2024 Apr 30;9(2):137–42. doi: 10.5194/jbji-9-137-2024 (PMC11184614; doi:10.5194/jbji-9-137-2024)
Supplement: The supplement related to this article is available online at: https://doi.org/10.5194/jbji-9-137-2024-supplement. [file jbji-9-137-supplement.zip › Table S1.pdf]

Table S1 Characteristics of the studies included in the literature review, including Authors information and Article data.

First Author	Year	Country	Journal	Joint	Tot. Patients	PJI patients	Male%
Browning	2022	Australia	Journal of Bone and Joint Infection	Knee, Hip, Shoulder	55	55	41.8
Lenguerrand	2019	UK	The Lancet - Infectious Diseases	Knee	3.659	3659	57,30%
Tsaras	2012	USA	Infection control and Hospital Epidemiology	Knee, Hip	7.375	70	38,60%
Walocha	2022	USA	Journal of Orthopedics	Shoulder	51.824	879	47,70%
Massin	2016	France	Knee Surgery Sports Traumatology Arthroscopy	Knee	285	285	47%
Tayton	2016	New Zealand	Bone and Joint Journal	Knee	64.566	106	63,20%
Keemu	2023	Finland	Acta Orthopaedica	Knee	620.087	484	54%
Castano-Betancourt	2018	Brazil	Journal of Orthopaedic Surgery and Research	Knee, Hip	598	38	n.a.
Wimmer	2016	Germany	Acta Orthopaedica Belgica	Knee, Hip	120	n.a.	n.a.

n.a. not available
